# Efficacy and safety of tirzepatide in patients with type 2 diabetes mellitus: A bayesian network meta-analysis

**DOI:** 10.3389/fphar.2022.998816

**Published:** 2022-10-14

**Authors:** Ruifang Guan, Qing Yang, Xiaolei Yang, Wandi Du, Xuening Li, Guo Ma

**Affiliations:** ^1^ Department of Clinical Pharmacy, School of Pharmacy, Fudan University, Shanghai, China; ^2^ Department of Clinical Pharmacology, Zhongshan Hospital, Fudan University, Shanghai, China

**Keywords:** tirzepatide, type 2 diabetes mellitus, network meta-analysis, efficacy, safety

## Abstract

**Background:** In light of clinical trials comparing different doses of tirzepatide with selective glucagon-like peptide-1 receptor agonist (GLP1-RA) or insulin analogue, a bayesian network meta-analysis was conducted to investigate the efficacy and safety of tirzepatide in patients with type 2 diabetes mellitus (T2DM).

**Methods:** We systematically searched PubMed, Embase, Web of science, Cochrane Central Register of Controlled Trials, and ClinicalTrials.gov from their inception to 2 May 2022. Final included studies met the eligibility criteria and methodological quality recommendations. Data analysis was performed using Stata 15.1 software. Each outcome was presented as a mean difference or an odds ratio, and the surface under the cumulative ranking curve value (SCURA).

**Results:** Ultimately, eight eligible RCTs involving 7245 patients were included. Generally speaking, compared with basal insulin (glargine or degludec); selective GLP1-RA (dulaglutide or semaglutide once weekly), 10 and 15 mg of tirzepatide exhibited better antidiabetic and weight-loss effect, especially, 15 mg of tirzepatide was dominant on reducing glycated hemoglobin (SCURA probability: 93.5%), body weight (99.7%), and fasting serum glucose (86.6%). As for safety, insulin caused less gastrointestinal events (93.5%), and there was no statistical difference between GLP1-RA and tirzepatide.

**Conclusion:** Compare with insulin and GLP1-RA, tirzepatide display favorable efficacy and acceptable safety for T2DM patients. More well-designed RCTs are needed to evaluate its clinical performance with higher doses of GLP1-RA and determine its potential cardiovascular benefits.

## 1 Introduction

Type 2 diabetes mellitus (T2DM) is a complex metabolic disorder, which not only characterized by hyperglycemia, but also associated with insulin resistance, impaired insulin secretion, hypertension, dyslipidemia and so on (Ferrannini and Cushman, 2012; DeFronzo et al., 2015; Taskinen and Borén, 2015). The International Diabetes Federation (IDF) estimated there were 537 million patients with diabetes around the world in 2021, and it will reach 783 million by 2045 (Sun et al., 2021). Many risk factors could lead to increased risk for T2DM, e.g., genetic history, unhealthy lifestyle, and obesity. Obesity is believed to be a strong promoter (Malone and Hansen, 2019), and it was also proved that T2DM interlinked with obesity in a pathophysiological way (Pappachan et al., 2019). Poor management of T2DM and obesity will increase the risk of cardiovascular disease (CVD). Some evidence has verified the importance of obesity in the progression of T2DM and CVD, and the association between weight-loss and long-term decrease in cardiovascular risk (Lavie et al., 2009). Thus, ideal medication of T2DM should be efficacious in lowering glucose as well as promoting weight loss, which have proven cardiovascular benefits and low risk of adverse events (Min and Bain, 2021).

Glucagon-like peptide one receptor agonists (GLP1-RAs) have demonstrated remarkable glycemic control, favorable weight-loss, and cardiorenal outcomes, thus are recommended as first-line injectable therapy for T2DM patients by current guidelines (Draznin et al., 2022). Glucose-dependent insulinotropic polypeptide (GIP) is another incretin hormone, is responsible for the amplification of insulin secretion, and regulates glucose homoeostasis together with glucagon-like peptide 1 (GLP-1) (Nauck and Meier, 2016). Yet, unlike GLP1, GIP does not inhibit appetite or food intake (Holst and Rosenkilde, 2020), and provides potential protection against hypoglycemia (Christensen et al., 2011). Moreover, GLP1 and GIP may improve β-cells functionality through synergistical pharmacological activation (Bastin and Andreelli, 2019). Therefore, GIP-based therapy has become an attractive candidate to be combined with GLP1-RAs (Papachristou et al., 2021). As a novel GLP1/GIP receptor co-agonist allowing once-weekly subcutaneous administration, tirzepatide was developed in this context.

According to a published systematic review, tirzepatide showed robust reductions of glycated hemoglobin (HbA1c, −1.94%), fasting serum glucose (FSG, −54.72 mg/dl) and body weight (−8.47 kg). It was a safety profile similar to long-acting GLP1-RAs (Bhagavathula et al., 2021). Up to now, there are two reported Phase 2 clinical trials and six Phase 3 clinical trials (SURPASS 1-5, J-mono) to compare the efficacy and safety of tirzepatide with placebo or active comparators (i.e., semaglutide (Frías et al., 2021), dulaglutide (Frias et al., 2018; Inagaki et al., 2022), basal insulin analogue degludec (Ludvik et al., 2021) and glargine (Del Prato et al., 2021; Dahl et al., 2022)). In these studies, the weight-loss and glucose-lowering effect was greater than the comparators. The gastrointestinal tolerance of tirzepatide was comparable to the GLP1-RAs. Network meta-analysis is a well-established approach which allows the available comparison of a complete set of interventions so as to assess their comparative efficacy and safety (Laws et al., 2019). Accordingly, we conducted a network meta-analysis to systematically estimate the efficacy and safety of tirzepatide to provide basis for its future use.

## 2 Methods

This systematic review conformed to PRISMA (Preferred Reporting Items for Systematic Reviews and Meta-Analysis) (Moher et al., 2009) and its extension for the network meta-analyses (Hutton et al., 2015).

### 2.1 Search strategy

The literature search was conducted in PubMed, Embase, Web of Science, Cochrane Central Register of Controlled Trials, and ClinicalTrials.gov from their inception to 2 May 2022 without language restriction. The databases were searched with the following MeSH (Medical Subject Headings) terms or keywords: 1) “diabetes mellitus, type 2” OR “diabetes mellitus, type II” OR “noninsulin dependent diabetes” OR “non-insulin dependent diabetes” OR “NIDDM” OR “type II diabetes” OR “type 2 diabetes” OR “T2DM” OR “mature onset diabetes” OR “late onset diabetes” OR “adult onset diabetes”; AND 2) “tirzepatide” OR “LY3298176”. Furthermore, the reference lists from the retrieved articles were screened to search for additional relevant studies.

### 2.2 Study selection

Studies were included in the systematic review if they met all of the following criteria: 1) Randomized controlled trials (RCTs) comparing tirzepatide with placebo or active therapeutic interventions in the patient with T2DM; 2) Minimum intervention period of 12 weeks; 3) Investigating the efficacy of tirzepatide on blood glucose parameters or body weight and its safety profile. Secondary analyses and phase I studies were excluded. Two reviewers, working independently, screened citations and evaluated full text records for eligible studies. Discordances were discussed with the third reviewer and resolved by consensus.

### 2.3 Data extraction and quality assessment

For each eligible study, two reviewers (RG and QY) independently extracted the following information: study characteristics (year of publication, study sites, study design), population (sample size, patient demographics, duration of disease; baseline HbA1c), description of interventions (drug class, name, dose), duration of treatment, and measured outcomes. The reviewers resolved disagreements by discussion or consulting the third reviewer (XY).

Since 1 mg was far below the effective dose, and only 29 participants who received 12 mg tirzepatide, these two dosing regimens were excluded. Three doses (5 mg, 10 mg, 15 mg) were included in the meta-analysis. Efficacy outcomes consisted of the mean changes in glycated hemoglobin (HbA1c), fasting serum glucose (FSG), and body weight. The number of gastrointestinal adverse events was chosen to represent safety outcomes. For all the outcomes, we extracted data for the modified intention-to-treat (mITT) population, which was defined as all the randomly assigned participants who received at least one dose. We assessed the risk of bias for all the included RCTs with the Cochrane risk of bias tool (Sterne et al., 2019), which includes random sequence generation, allocation concealment, blinding, missing outcome data, and selective reporting of outcomes.

### 2.4 Data synthesis and analysis

A network meta-analysis was performed with Bayesian approach using Stata 15.1 (Stata Corp, College Station, TX, United States) in the present study. Effect estimates included odds ratio (OR) for dichotomous outcomes and mean difference (MD) for continuous outcomes. Semaglutide and dulaglutide were combined in a single group (GLP1-RA), and insulin degludec and glargine in another group (Insulin). Point estimates and 95% confidence interval (CI) were assessed using Markov Chain Monte method in Stata with a random-effects model. The surface under the cumulative ranking curve (SUCRA) was also calculated to rank the effectiveness of each treatment. The SUCRA was used to estimate the ranking probabilities for different interventions. A higher SUCRA value indicates a better treatment. Cochran Q test and I^2^ statistic were used to assess heterogeneity levels, agreement between direct and indirect estimates in every closed loop of evidence using node splitting approaches. *p*-value was used to test the degree of inconsistency. When *p* > 0.05, the heterogeneity is not obvious, and the difference within a group is considered as small. Publication bias was evaluated using funnel plots.

## 3 Results

### 3.1 Study selection and characteristics

**FIGURE 1:**
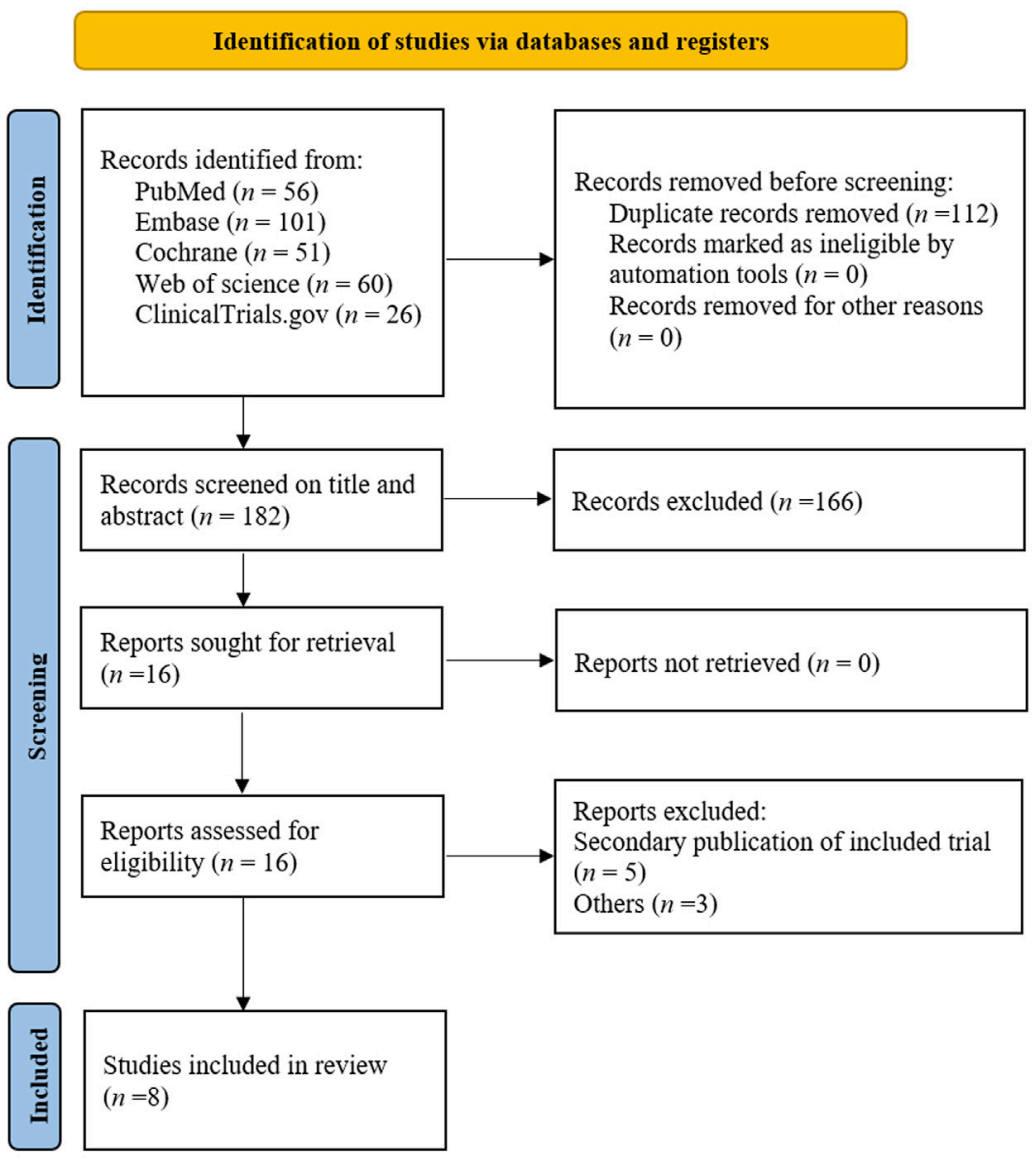
PRISMA flowchart of literature search for eligible studies.

The electronic search yielded 294 unique records. After removal of 112 duplicate records and screening 182 titles and abstracts, 16 reports were left for full-text assessment. After further selection (Figure 1), eight unique RCTs fulfilled the inclusion criteria (Frias et al., 2018; Frias et al., 2020; Del Prato et al., 2021; Frías et al., 2021; Ludvik et al., 2021; Rosenstock et al., 2021; Dahl et al., 2022; Inagaki et al., 2022). RCTs were published between 2018 and 2022 including 7245 participants (range, 111–1995 participants) with T2DM in total; six (75%) of them were multinational RCTs. Weighted means of baseline HbA1c, weight, and age were 8.2%, 90.3 kg, and 57.9 years, respectively; the duration of interventions ranged from 12 to 52 weeks. The detailed characteristics of individual study are provided in Supplementary Table S1.

**FIGURE 2:**
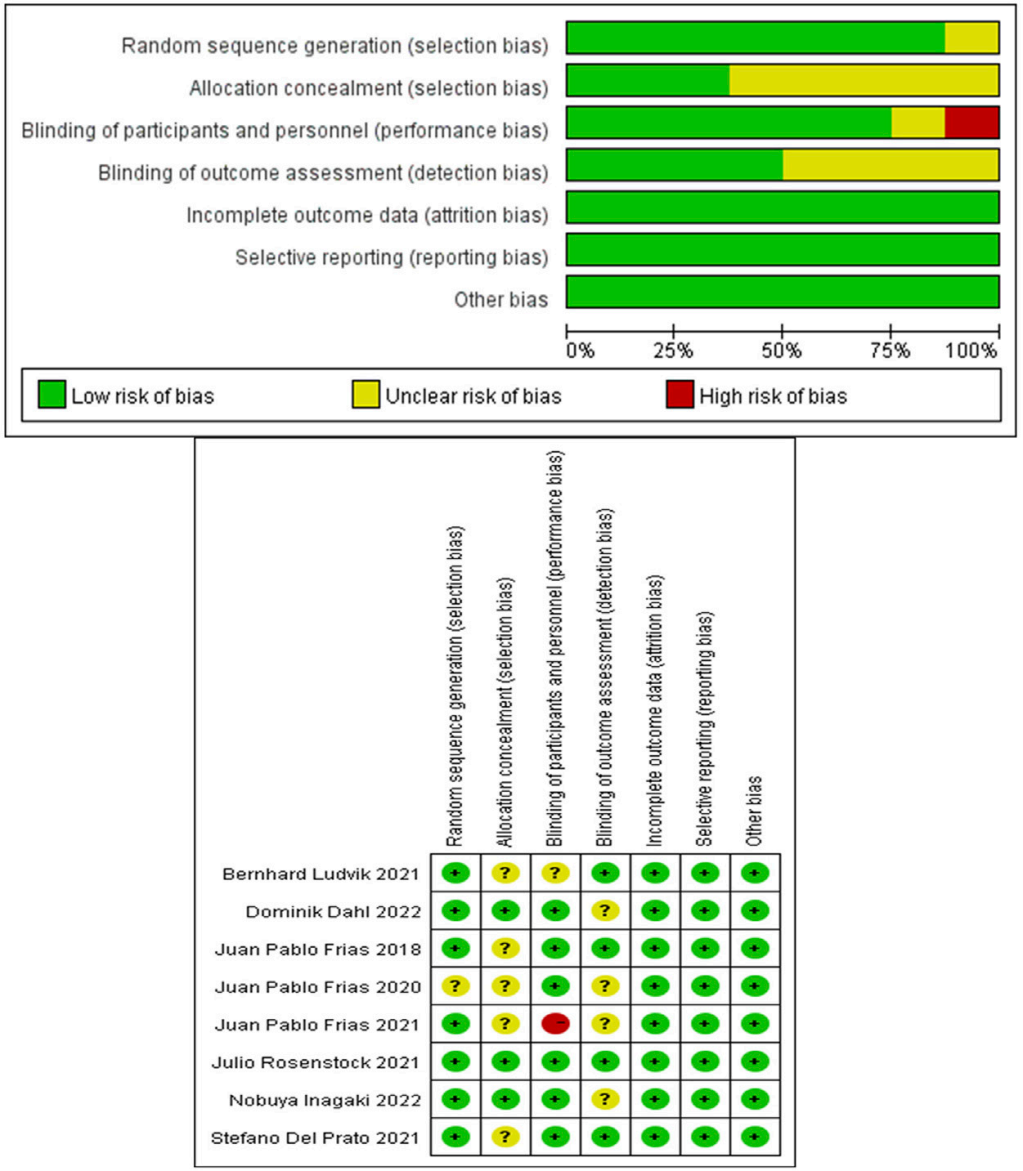
Assessment of the risk of bias in included studies.

The risk of bias in eligible RCTs was shown in Figure 2. Among the eight RCTs, all of them had low risk for bias of incomplete outcome data and selective reporting, seven RCTs had low risk for bias in random sequence generation, five RCTs had unclear risk for bias in allocation concealment, six RCTs had low risk for bias in blinding participants and personnel, and four RCTs had low risk for bias in blinding the outcome assessment. One study was deemed to possess high performance risk because it did not provide details of blinding the participants (Rosenstock et al., 2021). Overall, these studies had a low or moderate level of risk.

### 3.2 Network meta-analyses

**FIGURE 3:**
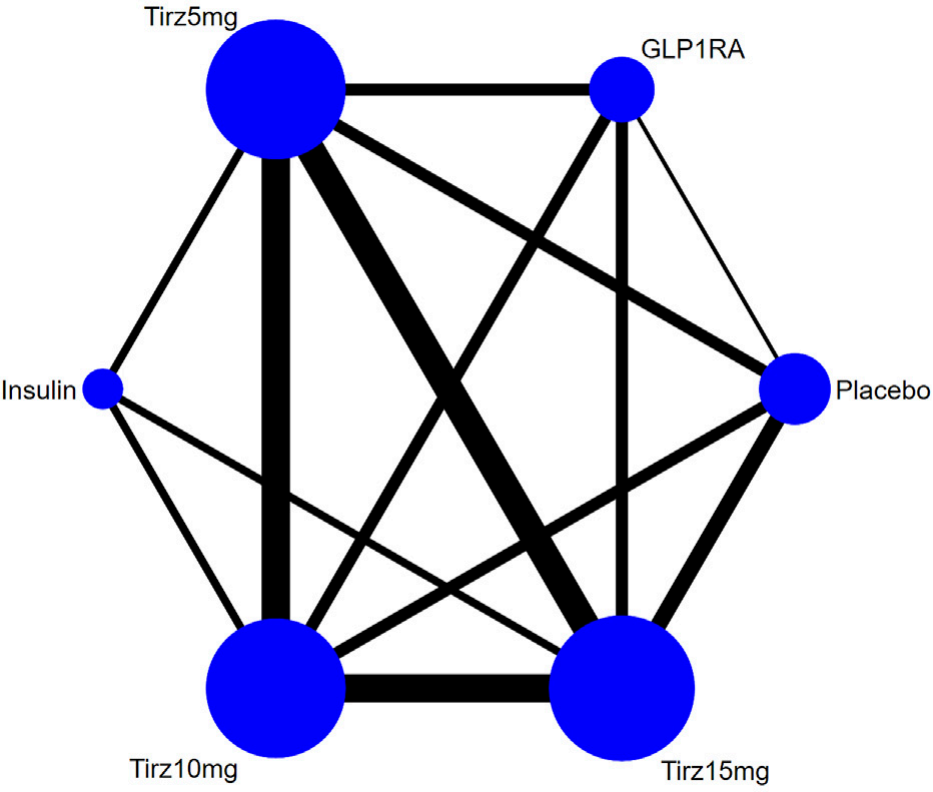
Network plot of effectiveness and safety outcomes. Note: The width of the lines in the network graph is proportional to the number of RCTs, and the node sizes correspond to the number of randomly assigned participants in the treatment comparisons. Tirz stands for tirzepatide. Semaglutide and dulaglutide are combined in group GLP1RA, and Insulin consists of degludec and glargine.

The data on HbA1c, FSG, body weight and safety profile were available from all the eight RCTs. There was no evidence of global inconsistency in any network. All *p*-values > 0.05, indicating the heterogeneity was not obvious. The treatment network was illustrated in Figure 3.

#### 3.2.1 HbA1c

**FIGURE 4:**
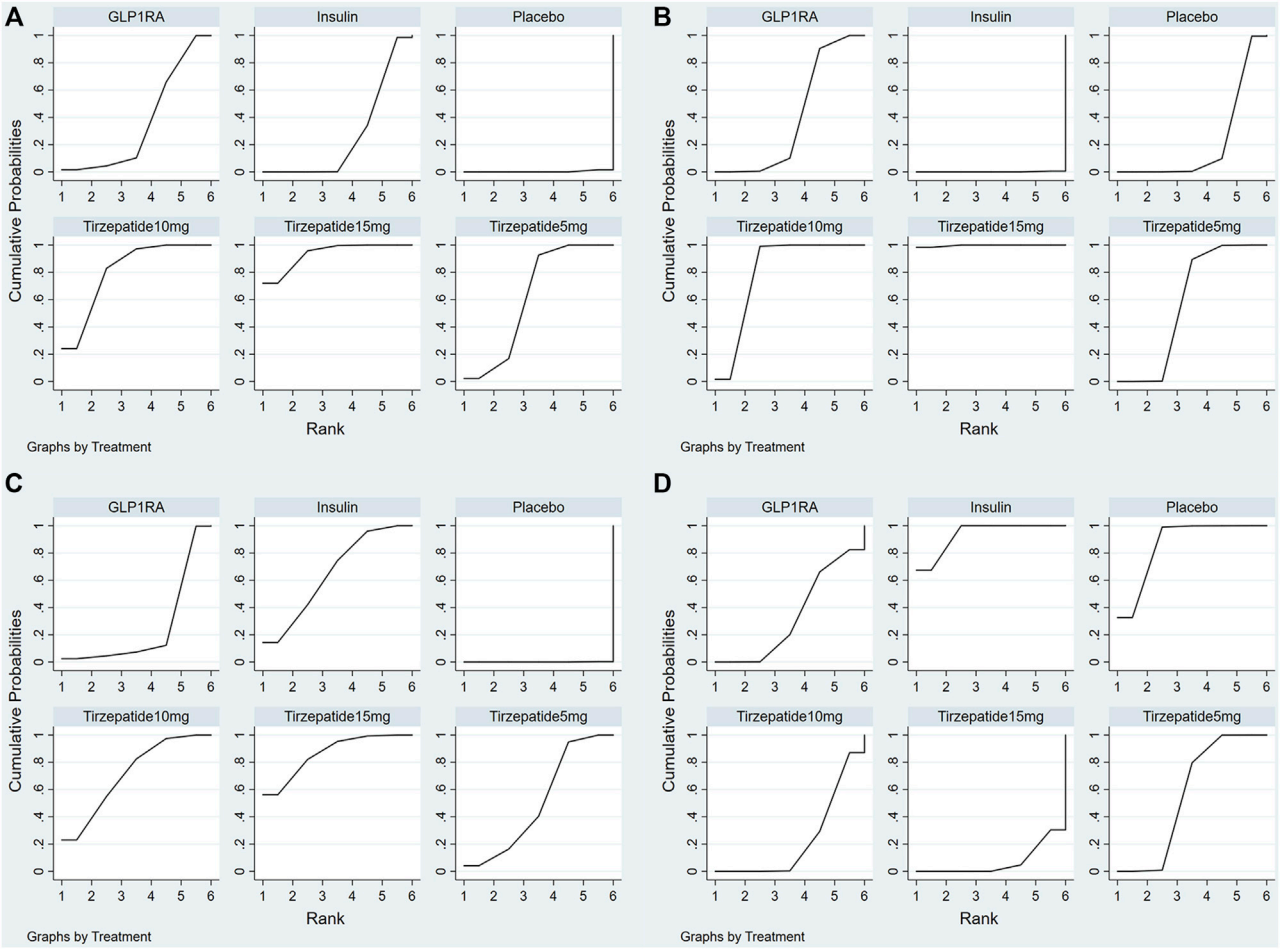
Plot of the surface under the cumulative ranking curves for outcomes. **(A)** HbA1c; **(B)** Body weight; **(C)** FSG; **(D)** gastrointestinal adverse events. Note: The surface under the cumulative ranking curve (SUCRA) was used to estimate the ranking probabilities for different treatments, which ranged from 0% to 100%. A better treatment is indicated by a higher SUCRA value.

The results suggested that, compared with Insulin, all the doses of tirzepatide exhibited statistically considerable improvements in reducing HbA1c. 10 mg and 15 mg of tirzepatide were more effective than GLP1-RA. (Table 1). The SUCRA was used to estimate the ranking probabilities for different interventions. A higher SUCRA value indicates a better treatment. Figure 4A and Table 2 presented the SUCRA values of different interventions. 15 mg of tirzepatide was associated with the highest probability of being the best option for the reduction of HbA1c (93.5%), followed by 10 mg of tirzepatide (80.9%), 5 mg of tirzepatide (62.4%), GLP1-RA (36.4%), Insulin (26.5%), and placebo (0.3%).

**TABLE 1:** Results of the network meta-analysis of the HbA1c (upper right quarter) and the body weight (lower left quarter).

Tirzepatide 5 mg	−0.80 (−1.57, −0.03)	−0.40 (−1.17,0.37)	−0.13 (−1.03,0.78)	0.70 (0.24,1.15)	0.50 (−0.26,1.26)
6.50 (3.03,9.97)	**Tirzepatide 15 mg**	0.40 (−0.38,1.18)	1.70 (0.93,2.47)	1.50 (0.60,2.40)	1.30 (0.53,2.07)
3.90 (0.51,7.29)	−2.60 (−6.09,0.89)	**Tirzepatide 10 mg**	0.11 (−0.78,0.99)	1.10 (0.20,1.99)	0.90 (0.13,1.67)
−2.26 (−6.28,1.76)	−4.40 (−7.80, −1.00)	−1.45 (−5.36,2.46)	**Placebo**	−1.00 (−1.90, −0.11)	−1.20 (−1.97, −0.43)
−9.37 (−11.33, −7.41)	−15.87 (−19.85, −11.89)	−13.27 (−17.19, −9.36)	−4.97 (−8.89, −1.05)	**Insulin**	−0.20 (−1.08,0.69)
−2.10 (−5.47,1.27)	−8.60 (−12.08, −5.12)	−6.00 (−9.40, −2.60)	2.30 (−1.11,5.71)	7.27 (3.37,11.17)	**GLP1-RA**

The differences between the compared groups were deemed as significant when the 95% CI, did not contain 0.00, which is marked with grey background.

**TABLE 2:** Ranking probability of various interventions.

Intervention	HbA1c		Body weight		FSG		Safety	
	SUCRA (%)	Rank	SUCRA (%)	Rank	SUCRA (%)	Rank	SUCRA (%)	Rank
Placebo	0.3	6	21.9	5	0.1	6	86.3	2
Insulin	26.5	5	0.1	6	65.4	3	93.5	1
GLP1-RA	36.4	4	40.2	4	25.2	5	33.8	4
Tirzepatide 5 mg	62.4	3	57.9	3	51.1	4	56.1	3
Tirzepatide 10 mg	80.9	2	80.1	2	71.6	2	23.3	5
Tirzepatide 15 mg	93.5	1	99.7	1	86.6	1	7.0	6

#### 3.2.2 Body weight

As shown in Table 1, compared with Insulin, all the doses of tirzepatide presented higher effectiveness in controlling body weight. Compared with GLP1-RA, 10 mg and 15 mg of tirzepatide showed statistically significant reductions in body weight. As shown in Table 2, 15 mg of tirzepatide seemed to be the best intervention for controlling body weight with a SUCRA value of 99.7%, followed by 10 mg of tirzepatide (80.1%), 5 mg of tirzepatide (57.9%), GLP1-RA (40.2%), placebo (21.9%), and Insulin (0.1%) (Figure 4B).

#### 3.2.3 FSG

The results indicated that with respect to reducing FSG, there were no differences between all the doses of tirzepatide and Insulin; compared with GLP1-RA, 10 mg and 15 mg of tirzepatide demonstrated more favorable effect (Table 3). The results of the SUCRA analysis (Table 2) showed that 15 mg of tirzepatide (86.6%) seemed to be the most effective option for significantly reducing FSG, followed by 10 mg of tirzepatide (71.6%), Insulin (65.4%), 5 mg of tirzepatide (51.1%), GLP1-RA (25.2%), and placebo (0.1%) (Figure 4C).

**TABLE 3:** Results of the network meta-analysis of the FSG (upper right quarter) and gastrointestinal adverse events (lower left quarter).

Tirzepatide 5 mg	−0.90 (−2.49,0.69)	−1.10 (−2.66,0.46)	−1.16 (−2.99,0.66)	−0.22 (−1.10,0.66)	1.10 (−0.46,2.66)
0.25 (0.09,0.67)	**Tirzepatide 15 mg**	−0.20 (−1.79,1.39)	3.20 (1.63,4.77)	0.68 (−1.14,2.49)	2.00 (0.40,3.60)
0.47 (0.18,1.24)	1.87 (0.70,4.98)	**Tirzepatide 10 mg**	−0.17 (−1.97,1.63)	0.88 (−0.91,2.66)	2.20 (0.64,3.76)
0.63 (0.16,2.46)	4.48 (1.31,15.25)	2.31 (0.77,6.91)	**Placebo**	−3.42 (−5.23, −1.62)	−2.10 (−3.68, −0.52)
6.00 (3.67,9.81)	23.97 (7.96,72.20)	12.82 (4.30,38.24)	1.34 (0.36,5.02)	**Insulin**	1.32 (−0.47,3.12)
0.66 (0.25,1.73)	2.62 (0.99,6.94)	1.40 (0.54,3.67)	0.15 (0.04,0.49)	0.11 (0.04,0.32)	**GLP1-RA**

The differences between the compared groups were deemed as significant when the 95% CI, did not contain 0.00 (upper right quarter) or 1.00 (lower left quarter), which is marked with grey background.

#### 3.2.4 Safety outcome

The number of gastrointestinal adverse events, which includes nausea, vomiting and diarrhea, was chosen to summarize the overall safety in this network meta-analysis. As shown in Table 3, compared with GLP1-RA, there were no significant differences on the safety profile of all the doses of tirzepatide. All the dosing regimens of tirzepatide caused more gastrointestinal adverse events than Insulin. The superiority of Insulin (93.5%), placebo (86.3%) and 5 mg of tirzepatide (56.1%) in overall safety was further confirmed by the SUCRA analysis (Figure 4D and Table 2). The risk of gastrointestinal adverse events increased along with increase of dose.

### 3.3 Publication bias

**FIGURE 5:**
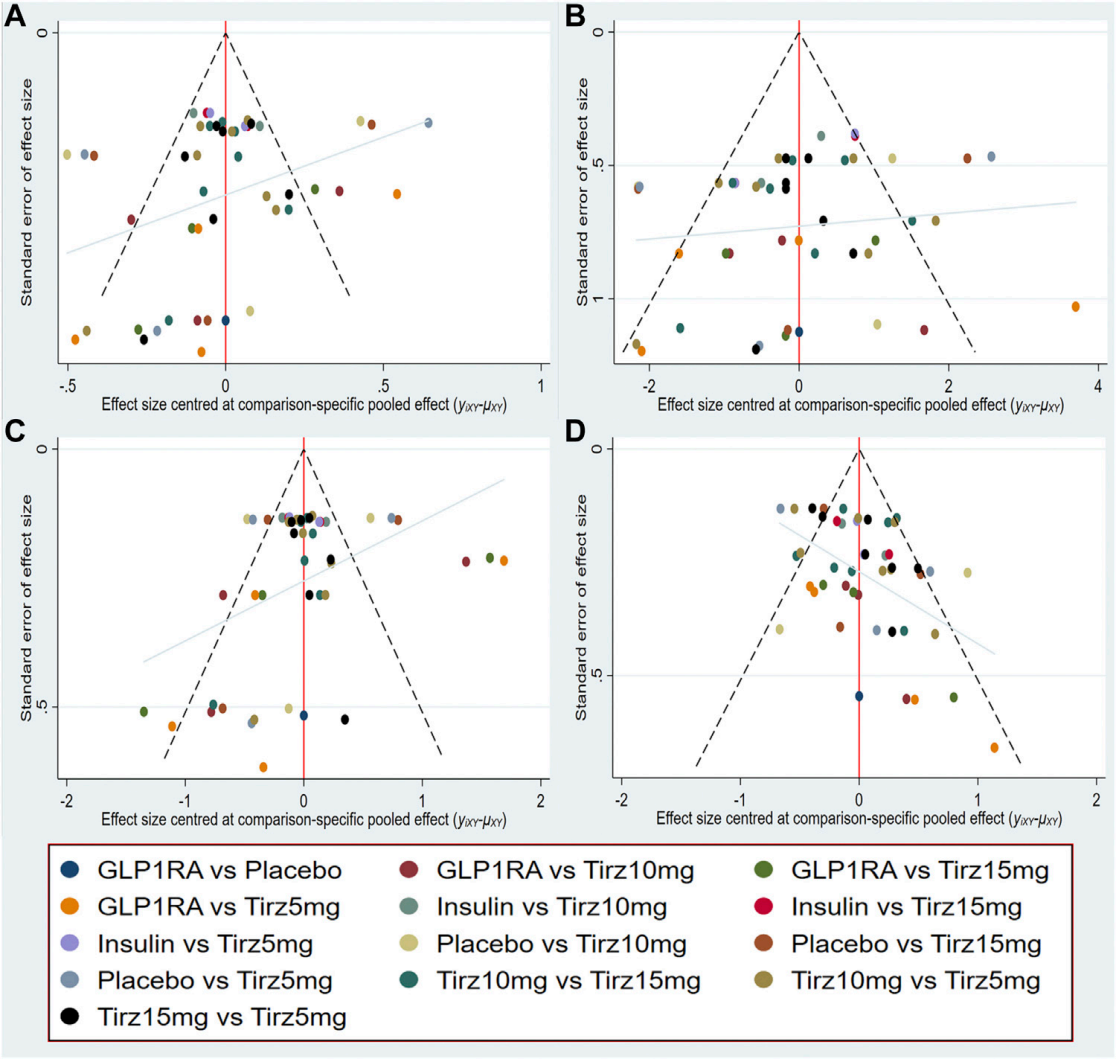
Comparison-adjusted funnel plot for **(A)** HbA1c; **(B)** Body weight; **(C)** FSG; **(D)** gastrointestinal adverse events.

Publication bias was measured by the comparison-adjusted funnel plots in this network meta-analysis. Visual inspections indicated that distribution of the included RCTs were relatively symmetrically distributed based on the vertical zero line, and there was some angle between the adjusted auxiliary line and the horizontal zero line. Therefore, some publication bias may exist (Figure 5).

## 4 Discussion

GIP was not considered as an appealing target for the treatment of T2DM, because early research showed there was no reduction of GIP secretion in patients with diabetes, and GIP agonism seemed to have a less insulinotropic effect (Fisman and Tenenbaum, 2021). Until recently years, GIP agonism were proved effective under better glycemic control (Højberg et al., 2009). It could not only enhance meal-stimulated insulin secretion, but also modulate glucagon secretion in a glucose-dependent manner to facilitate postprandial lipid clearance and regulate food intake (Coskun et al., 2022). In addition, GIP amplifies adipose-tissue sensitivity to insulin, as well as enhancing GLP1-mediated central satiety (Nauck et al., 2021). Since glucagon can reduce body weight and appetite, delay gastrointestinal transit, and stimulate insulin secretion (Dailey and Moran, 2013). Thus, as a regulator of glucagon, GIP/GLP1 dual agonists provide bigger weight reduction than GLP1-RAs. Tirzepatide was developed based on those evidence. It was proved that the effects of tirzepatide improved biomarkers of β-cell function and insulin resistance (Thomas et al., 2021).

Our network meta-analysis identified eight RCTs that randomized T2DM patients to six interventions, including selective GLP-1RA (semaglutide and dulaglutide), basal insulin analogue (degludec and glargine), three different doses of tirzepatide (5 mg, 10 mg, 15 mg), and placebo. In terms of efficacy outcomes, compared with Insulin or GLP1-RA, high doses (10, 15 mg) of tirzepatide showed robust effectiveness in reducing HbA1c, body weight and FSG. As for safety, tirzepatide had similar safety profile with GLP1-RA. Comparing with Insulin, it may increase gastrointestinal adverse events. The GLP1-RA group in the network meta-analysis contained 0.75 mg and 1.5 mg of dulaglutide, and 1 mg of semaglutide, yet higher dosing of regimen had already either received marketing approval (dulaglutide 3 mg and 4.5 mg) or received recommendations of authority (semaglutide 2 mg) (Karagiannis et al., 2022). The comparison of efficacy and safety between tirzepatide and higher doses of GLP1-RA is unclear, thus further RCTs are needed. According to the regulatory guidance of US Food and Drug Administration (FDA), hypoglycemic agents are required to demonstrate cardiovascular safety. Yet, to date, only SURPASS-4 reported that tirzepatide was not associated with excess cardiovascular risk (Del Prato et al., 2021). Another ongoing clinical trial SURPASS-CVOT (NCT04255433) will provide more data on comparing the cardiovascular outcomes of tirzepatide with dulaglutide which has demonstrated cardiovascular benefits in T2DM patients with high cardiovascular risk (Gerstein et al., 2019). Now, management of obesity is believed as a primary treatment goal of T2DM, and weight-loss is in favor of reversing the underlying metabolic abnormalities of T2DM (Lingvay et al., 2022). Tirzepatide showed robust weight-loss effect even when it was used in conjunction with insulin glargine, which was associated with weight-gain. Tirzepatide seems to be a novel and promising therapeutic option for the management of T2DM, obesity and its related cardiometabolic diseases. In addition to better health outcomes and fewer adverse effects, the patients with T2DM may prefer to minimizing multiple insulin injections, avoiding hypoglycemia, lowering weight or reducing risk of weight gain. Tirzepatide exactly display these characteristics and advantages.

There were several published meta-analyses of tirzepatide (Bhagavathula et al., 2021; Dutta et al., 2021; Karagiannis et al., 2022; Sattar et al., 2022), which only included six or seven RCTs. Our article was the first bayesian network meta-analysis of tirzepatide including eight RCTs, and first combined the participants who received the same dose from different RCTs in one group, then compared and evaluated the relative efficacy and safety of different doses of tirzepatide with Insulin and selective GLP1-RA. In the present study, a sensitive search comprehensibly covering the latest research findings, independent study identification, selection and data extraction was performed by two reviewers. It needs to be noted that, heterogeneity in clinical settings, enrolled population, and follow-up duration cannot be avoided. Moreover, publication bias may exist. Especially, as a brand-new drug, at present there are very few RCTs of tirzepatide. In the future, more multicenter RCTs of tirzepatide will provide more information of this promising hypoglycemic drug.

## 5 Conclusion

In summary, efficacy and safety of tirzepatide were systematically evaluated based on the available eight RCTs in this bayesian network meta-analysis. Compared with the marketed antidiabetic drugs (e.g., Insulin, GLP1-RA), tirzepatide exhibited robust efficacy in reducing HbA1c, body weight, and FSG with an acceptable safety profile. In the future, effectiveness, safety, economics, suitability and accessibility of tirzepatide as well as its potential cardiovascular benefits should be further evaluated with evidence-based medicine and real-world evidence.

## Abbreviations

GLP1-RA, glucagon-like peptide-1 receptor agonist; T2DM, type 2 diabetes mellitus; SCURA, the surface under the cumulative ranking curve value; GIP, glucose-dependent insulinotropic polypeptide; HbA1c, glycated hemoglobin; FSG, fasting serum glucose; RCT, randomized controlled trials; OR, odds ratio; CI, confidence interval.
